# Acute Poisoning in Children Presenting to the Pediatric Emergency Department: An Epidemiologic Study and the Impact of the SARS-CoV-2 Pandemic

**DOI:** 10.3390/medicina61091507

**Published:** 2025-08-22

**Authors:** Lorenzo Di Sarno, Valeria Pansini, Anya Caroselli, Paolo Maurizio Soave, Antonio Gatto, Serena Ferretti, Lavinia Capossela, Antonio Chiaretti

**Affiliations:** 1 Institute of Pediatrics, Fondazione Policlinico A. Gemelli IRCCS, 00168 Rome, Italy; lorenzo.disarno1@guest.policlinicogemelli.it (L.D.S.); valeria.pansini@policlinicogemelli.it (V.P.); serena.ferretti1@guest.policlinicogemelli.it (S.F.); lavinia.capossela1@guest.policlinicogemelli.it (L.C.); 2 Institute of Pediatrics, Fondazione Policlinico A. Gemelli IRCCS, Università Cattolica del Sacro Cuore, 00168 Rome, Italy; anya0196@gmail.com (A.C.); antonio.chiaretti@policlinicogemelli.it (A.C.); 3 Department of Emergency, Anesthesiology and Resuscitation Sciences, Fondazione Policlinico Universitario “A. Gemelli” IRCCS, 00168 Rome, Italy; paolomaurizio.soave@policlinicogemelli.it

**Keywords:** children, pediatric, poisoning, toxicology

## Abstract

*Background and Objectives*: Acute poisoning is a frequent cause of pediatric emergency department visits and represents a significant public health issue, with children particularly vulnerable due to developmental and behavioral factors. This study aimed to characterize the clinical and demographic profiles of pediatric patients presenting with acute intoxication over a ten-year period (2014–2023) and to assess the impact of the SARS-CoV-2 pandemic on patterns of pediatric poisoning. *Materials and Methods*: A retrospective cohort study was conducted at the Fondazione Policlinico A. Gemelli in Rome, including all patients under 18 years presenting with acute intoxication between September 2014 and June 2023. Data were extracted from electronic medical records and categorized by age group (0–5, 6–11, and 12–18 years) and by three pandemic-related periods: Pre-COVID-19 (to March 2020), COVID-19 (March 2020–June 2021), and Post-COVID-19 (June 2021 onwards). Statistical analyses included chi-squared tests and Cramér’s V for effect size. *Results*: Of 794 PED admissions for acute intoxication, 64.5% involved children aged 0–5 years, and 24.9% involved adolescents. Most events occurred at home (63.2%) and were accidental (76.4%), with voluntary intoxications exclusively among adolescents. Drug ingestion was the leading cause (39.3%), followed by solvents (17.8%) and alcohol (7.8%), with alcohol-related cases rising markedly post-pandemic. Statistically significant associations were found between the pandemic period and both age group and intentionality of poisoning (*p* < 0.00001), but not gender. Voluntary and recreational intoxications increased during and after the pandemic, particularly among adolescents. *Conclusions*: Pediatric acute poisoning displays a bimodal age distribution, with accidental exposures predominant in young children, and intentional cases predominant in adolescents. The COVID-19 pandemic was associated with an increase in adolescent voluntary and recreational intoxications. These findings underscore the need for targeted prevention strategies addressing both household safety for young children and mental health and substance use interventions for adolescents.

## 1. Introduction

In pediatric medicine, acute intoxications are a common reason for presentation to the pediatric emergency department (PED) and pose a notable public health concern [1]. Due to their developmental stage, behavior, and limited awareness of potential hazards, children are especially vulnerable to toxic exposures. Pediatric poisonings are commonly categorized by duration (acute or chronic), intentionality (accidental, pseudo-voluntary, or voluntary), and route of exposure, with ingestion being the most frequent [1,2]. Unintentional ingestion of household substances is particularly prevalent among children under five years old due to their exploratory behavior and the often-unsafe storage of these substances [3].

In adolescents, intoxications are more often intentional and may arise from various motivations ranging from recreational substance use to self-harming behaviors associated with psychological distress [4,5]. This age group typically has greater access to medications, alcohol, and narcotics, which are frequently employed as coping mechanisms in response to emotional vulnerability, social isolation, or underlying psychiatric conditions [6,7]. Additionally, environmental risk factors—particularly those present in the home environment—and inadequate preventive measures, such as improper storage or repackaging of hazardous substances, further exacerbate the incidence of these events. Common toxic agents implicated include pharmaceuticals, cleaning products, alcohol, and illicit substances [8,9]. The COVID-19 pandemic and quarantine reshaped daily habits and home environments by confining families indoors, disrupting routines, and increasing stress and anxiety. This led to more time spent at home, greater exposure to household substances, and heightened psychological distress, especially among children. Consequently, these changes plausibly contributed to shifts in pediatric acute intoxications seen in PED [10,11].

The primary objective of this study is to characterize the clinical and demographic profiles of pediatric patients presenting to the PED with acute intoxications over the ten-year period from 2014 to 2023. This includes a detailed analysis of the frequency and mode of intoxication, the types of substances ingested, treatment modalities employed, and patient outcomes. The secondary aim of this study is to determine the COVID-19 pandemic’s impact on pediatric acute poisoning. Specifically, we analyze statistically significant associations between pre-pandemic, pandemic, and post-pandemic periods and the clinical and demographic characteristics of affected children.

## 2. Materials and Methods

### 2.1. Study Design and Data

We conducted a retrospective cohort study at the Pediatric Emergency Department (PED) of the “Fondazione Policlinico Universitario Agostino Gemelli” in Rome, Italy. All patients aged <18 years presenting with acute intoxication between September 2014 and June 2023 were eligible. Ethical approval for this study was obtained from the Ethics Committee (ID 5995). The parents of the studied patients were informed about the purpose of this study and signed an informed consent form for consenting access to children’s medical records and for processing personal data. 

Patients were identified, and demographic, clinical, and management data were collected through electronic records on the internal information system for PED management (GIPSE^®^ platform). The GIPSE^®^ platform (“Gestione Informazioni di Pronto Soccorso Emergenza” = Emergency Department Information Management) is an information system used in the Italian region of Lazio, which includes the city of Rome, for emergency services and for automating the management of information flows. It supports users in managing emergency services and producing the documentation required by law [12]. Inclusion criteria were (1) confirmed or suspected acute intoxication due to ingestion, inhalation, mucocutaneous contact, or other exposition; (2) presentation within 24 h of exposure; and (3) availability of complete demographic and clinical data. Chronic poisonings, non-toxic foodborne illnesses, and incomplete records were excluded. The study population was categorized into three age groups: pre-school age (0–5 years), school age (6–11 years), and adolescence (12–18 years).

Regarding the SARS-CoV-2 pandemic, three distinct time periods were defined:

- The Pre-COVID-19 period, extending from the start of the study until 8 March 2020;
- The COVID-19 period, from 9 March 2020, to 21 June 2021, encompassing the initial lockdown and the subsequent easing of curfew measures;
- The Post-COVID-19 period, covering 22 June 2021, through the conclusion of the study.

### 2.2. Measures

The primary outcome was the occurrence of acute intoxication, classified by intentionality: accidental or voluntary (anticonservative vs. recreational). The primary independent variable was the pandemic period (Pre-COVID-19, COVID-19, and Post-COVID-19). Other covariates included the following:

- Demographics: age group and gender.
- Exposure context: environment (home, school, public place, and other); the presence of witnesses; and “time slot”—this last variable refers to the time of day when the patient was exposed to the toxic agent, as reported in the clinical record. To facilitate analysis, exposures were categorized into four 6 h intervals: 0–6, 6–12, 12–18, and 18–24. This categorization aimed to explore potential temporal patterns in exposure, which may be associated with variations in daily routines, supervision, and accessibility to toxic substances. The other exposure variables, including environment and presence of witnesses, were also considered to investigate potential patterns in exposure risk.
- Substance involved.
- Clinical presentation: signs and symptoms on arrival.
- Management: diagnostic tests performed, treatments administered, and outcome (discharge vs. admission; intensive vs. non-intensive care).

### 2.3. Statistical Methods

Descriptive statistics were used to summarize the study population. Categorical variables were described using frequency distribution (e.g., gender and age groups). Bivariate analysis was performed to assess associations between the primary independent variable (pandemic period) and categorical outcomes, using the chi-squared test (for categorical variables). A *p*-value < 0.05 was considered statistically significant.

Given the multiple comparisons conducted, the potential for Type I error inflation was considered; however, no formal adjustment for multiple testing was applied due to the exploratory nature of the analysis. Effect sizes for significant chi-square tests were calculated using Cramér’s V to quantify the strength of associations. Inferential analysis was limited to hypothesis testing for associations between categorical variables, as described above.

The statistical analysis was performed using STATA 18.0 software (Stata Corporation, College Station, TX, USA).

## 3. Results

During the study period, a total of 794 PED admissions for acute intoxication were documented. The pre-school age group was the most affected, accounting for 64.48% of cases; followed by adolescents, at 24.94%; and the school-age group, at 10.58%. Detailed features of the study population are presented in Table 1.

The most common setting for intoxication events was the home (63.22%), followed by public areas (8.44%), educational institutions (1.51%), and juvenile detention centers (0.38%). A specific location was not reported in 25.82% of cases. Incidents of intoxication were most frequent during the 18:00–24:00 (37.53%) and 12:00–18:00 (27.46%) time intervals. A wide amount of intoxications were classified as unintentional (76.45%), while a smaller percentage was attributed to voluntary intake (23.55%). No instances of intentional intoxication were observed in individuals below the age of 12.

Within the subset of voluntary intoxication cases, which were exclusive to adolescents, 60.43% were associated with anti-conservative behaviors, whereas 39.57% were related to recreational activities.

The primary clinical presentation in patients was asymptomatic (37.78%), with nausea and vomiting being the most frequently reported symptoms (23.30%), alongside pharyngeal and mucous membrane hyperemia (10.58%). Olfactory detection or physical evidence of the intoxicating substance was noted in 8.19% of patient evaluations.

**Figure 1:**
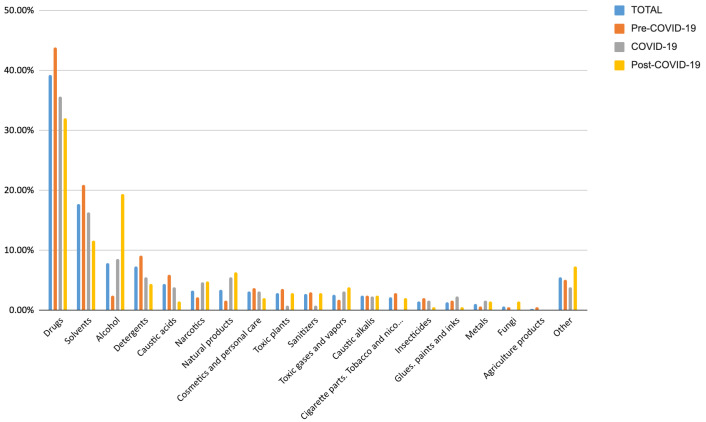
The overall incidence and the specific incidence of acute intoxications in distinct periods: Pre-COVID-19, COVID-19, and Post-COVID-19.

A comprehensive overview of the substances implicated in acute intoxication among pediatric patients—across the entire sample and within the pre-COVID-19, COVID-19, and post-COVID-19 periods—is shown in Figure 1 and detailed in Supplementary Table S1.

The primary cause of acute intoxication leading pediatric patients to access the PED was drug ingestion, accounting for 39.29% of cases in the total sample, with incidences of 43.79%, 35.66%, and 32.04% during the Pre-COVID-19, COVID-19, and Post-COVID-19 periods, respectively. Solvents were responsible for 17.76% of intoxications overall, peaking at 20.92% in the pre-COVID-19 period and subsequently declining to 16.28% and 11.65% in the COVID-19 and Post-COVID-19 periods. Intoxications due to alcohol and alcoholic beverages, with an overall incidence of 7.81%, demonstrated a marked increase from 2.40% in the Pre-COVID-19 period to 8.53% during COVID-19, rising further to 19.42% in the Post-COVID-19 period. Given that voluntary intoxication is an exclusively adolescent phenomenon, we sought to further analyze the substances involved in self-harm behaviors—predominantly drugs across all periods, peaking at 75.00% during the COVID-19 period, as detailed in Supplementary Table S2—and those used for recreational purposes, with alcohol being the most prevalent, followed by narcotics or combined use of both categories, as reported in Supplementary Table S3. Comprehensive information regarding the emergency management of pediatric patients with acute intoxication is provided in Supplementary Table S4. Among these patients, 52.39% underwent blood tests, while 30.23% received instrumental examinations. Esophagogastroduodenoscopy was performed in 5.16% of cases. The most common treatment administered was activated charcoal (15.24%), followed by gastric lavage (6.80%), with both treatments applied in 4.79% of cases. Antidotal therapy specific to the intoxicating substance was used in 1.51% of cases. The majority of patients (82.37%) were discharged following a variable observational period, whereas 17.63% required hospitalization; of these, 27.14% were admitted to intensive care units, and 72.86% were admitted to non-intensive care settings. Statistically significant correlations were observed between the analyzed time periods and age groups (*p* < 0.00001), as well as between the time periods and the intentionality of substance intake (*p* < 0.00001). Conversely, no significant correlation was identified between the time periods and patients’ gender (*p* = 0.536854), as detailed in Table 2.

Furthermore, significant associations were found between the analyzed periods and the incidence of intoxications with both self-harm intent (*p* = 0.000743) and recreational intent (*p* < 0.00001) across the entire sample, as illustrated in Table 2.

The strongest associations were between intentionality (V = 0.304) and recreational intent (V = 0.261) with the time periods, indicating moderate effects. Gender showed a very weak association, consistent with the non-significant *p*-value. Anti-conservative intent showed a weak association despite statistical significance.

## 4. Discussion

Poisoning represents a frequent and potentially life-threatening clinical condition in pediatric populations, with even mild exposure to xenobiotics constituting a distressing event for caregivers and a significant cause for emergency department referral [8]. The objective of this research was to examine the demographic and clinical characteristics of patients presenting to the PED as a result of poisoning incidents. The extensive and growing body of literature addressing this issue over recent years underscores the considerable healthcare burden it imposes [9,10,11,13,14,15].

Multiple studies consistently indicate that preschool children aged 1 to 5 years are the most susceptible pediatric age group to unintentional poisoning [16,17,18]. Kesapli et al. reported that the majority of poisoning incidents (55%) occur within the 0–5 years age group, with most cases being accidental [16]. Similarly, other research emphasizes that children aged 1–5 years account for approximately 80% of all poisoning cases observed in a pediatric emergency unit [17], whereas Saikia et al. stated that this age group is the most commonly affected in clinical profiles of childhood poisoning [18]. In contrast, older children and adolescents (13–17 years) tend to have poisoning cases related more to intentional self-harm rather than accidental exposures [19].

The observed trend in pediatric poisonings within this study aligns with existing literature, demonstrating two distinct peaks in incidence. We observed two prominent peaks, with the first and highest peak occurring during the preschool years (0–5 years), accounting for approximately 64.48% of cases. This heightened vulnerability in early childhood can be attributed to the natural curiosity of children and their limited awareness of potential hazards, which, when coupled with inadequate supervision, increases the likelihood of accidental ingestion or contact with xenobiotics commonly found in the home environment [20]. Additionally, this age group may be at risk due to parental medication errors or experiences of abuse and neglect, further exacerbating their susceptibility to harm [21]. Research underscores the critical need for preventive strategies and bespoke interventions to this developmental stage, given the profound impact of early exposure on neurodevelopment and behavior, including cognitive and emotional impairments observed in children prenatally exposed to substances [22].

The second, smaller peak in incidence, representing about 24.94% of cases, corresponds to adolescence, a developmental period marked by significant psychological complexity and vulnerability. Adolescents may engage in substance use as a form of recreational experimentation or as a coping mechanism to escape psychological distress [23]. This stage is also characterized by the emergence of mental health disorders, which can manifest as suicidal ideation or suicide attempts, highlighting the interplay between substance abuse and mental health challenges during adolescence [24]. The psychological fragility of this age group necessitates comprehensive mental health support and substance abuse prevention programs to mitigate risks and promote resilience.

This bimodal distribution of poisoning incidence—with a predominant peak in early childhood and a secondary peak in adolescence—reflects distinct developmental vulnerabilities [25].

Following the rapid and uncontrollable transmission of COVID-19, the Italian government was compelled to enforce a nationwide quarantine starting on 9 March 2020, alongside various public health interventions aimed at curbing the virus’s spread [26]. These interventions encompassed movement restrictions, closure of childcare facilities and schools, suspension of after-school programs, closure of playgrounds and public parks, and quarantine and isolation of suspected and confirmed cases [26]. Emerging research indicates that restrictive measures during the pandemic had profound adverse effects on children’s physical and mental well-being [27]. Subsequently, children experienced a different environmental exposure landscape compared to the pre-pandemic era. These evolving environmental and behavioral factors contributed to variances in childhood poisoning incidents [13]. Regarding the categories of xenobiotics analyzed in our study, drugs consistently represented the most common cause of intoxication from 2014 to 2023, primarily associated with household accidents among preschool-aged children and intentional poisonings during adolescence. Furthermore, alcohol consumption exhibited a marked exponential increase over the three specified periods, escalating from 2.40% during the pre-SARS-CoV-2 epidemic phase to 19.42% in the post-COVID-19 era. This increasing trend is in line with other studies in the literature that showed a significant boost—especially among girls—after the pandemic, especially when restrictions were eased or lifted [28]. According to our data, after the pandemic, there was a significant shift in demographic and intentionality profiles. Notably, the proportion of pre-school-aged children (0–5 years) decreased markedly from 73.2% pre-COVID-19 to 48.5% post-COVID-19, while adolescent cases (12–18 years) increased substantially from 15.0% to 42.2% over the same periods (*p* < 0.00001).

The increase in intoxication in adolescents in the Post-COVID-19 period could be related to the psychological impact of the pandemic, including stress, social isolation, and school-related difficulties [28]. These various factors may have contributed to a rise in the incidence of suicide attempts, as well as heightened susceptibility to substance use, which some individuals may have adopted as a maladaptive coping mechanism to manage elevated levels of stress, anxiety, and social isolation experienced during this period. Conversely, the observed reduction in cases of intoxication among pre-school-aged children throughout the COVID-19 pandemic is likely attributable to increased parental supervision and presence at home during lockdown measures, which may have limited young children’s access to potentially harmful xenobiotics [29]. Several studies analyzing pediatric poisoning cases demonstrate variable gender predominance depending on age and context. Large registry data from the ToxIC consortium showed male predominance in children under 6 years, with a reversal to female predominance in adolescents aged 13–18 years, where intentional pharmaceutical exposures were more common among females [30]. Hospital-based studies similarly report higher poisoning incidence in males overall, which was attributed to greater exploratory behavior in young boys [31]. However, some regional studies found female predominance, particularly in older children and adolescents, possibly reflecting sociocultural and behavioral factors influencing exposure and intent [32]. These findings showed that the gender distribution in pediatric poisonings could be age-dependent and influenced by the nature of the poisoning, underscoring the need for targeted prevention strategies considering sex and developmental stage. Conversely, in our study, gender did not show any prevalence, and there were no statistically significant differences in the intoxication trend between males and females during the three periods.

Intentionality also shifted dramatically, with accidental cases declining from 87.6% pre-COVID-19 to 58.3% post-COVID-19, and voluntary cases rising from 12.4% to 41.8% (*p* < 0.00001). Similarly, incidents involving recreational intent increased fivefold, from 3.5% to 21.4% (*p* < 0.00001), and those with anti-conservative intent rose significantly from 10.2% to 20.4% (*p* = 0.0007). These findings suggest a pronounced post-pandemic shift towards adolescent involvement and intentional, often recreational behaviors. This observation aligns with findings reported in recent literature. For instance, a 2024 cross-sectional observational study conducted by An et al. documented a marked rise in cases of intentional poisoning [9]. Specifically, the proportion of intentional poisoning admissions escalated from 34.5% in the pre-COVID-19 period to 58.7% during the COVID-19 pandemic [9]. The study by An et al. involved a comprehensive analysis of hospital records across multiple centers, highlighting the psychological and social stressors imposed by the pandemic as potential contributing factors to this increase [9]. One plausible hypothesis is that the psychological implications of the COVID-19 pandemic may have contributed to a marked increase in both suicide attempts and the recreational use and abuse of substances, including alcohol and narcotics. The observed exponential rise in cases of voluntary intoxication among adolescents serves as a critical indicator of deteriorating mental health within this vulnerable population [23]. This trend, when considered alongside the documented escalation in alcohol consumption, strongly implies an upsurge in emotional distress and the prevalence of psychiatric conditions such as anxiety and depression [33]. Moreover, these patterns suggest that affected individuals may be engaging in substance use as an immediate, albeit maladaptive, coping mechanism to mitigate the overwhelming psychological burden imposed by the pandemic [34]. Similarly, a Canadian study by Zhang et al. in 2022 examined PED visits for poisonings before and during the COVID-19 pandemic [10]. Despite an overall drop in PED visits, rates of poisonings increased significantly during the pandemic: recreational drug use-related visits rose by 160%, and intentional self-harm poisonings increased by 104.2% [10]. Increases were also seen in cannabis, vaping, and multi-substance poisonings. Hospital admissions for poisonings rose by 44.3% [10].

While our findings show an increased proportion of voluntary drug intoxications among adolescents during the COVID-19 and post-COVID-19 periods, this pattern should be interpreted while taking a few considerations into account. As shown in Table 2, the absolute number of intoxications decreased over the study period; however, this comparison is influenced by the fact that the pre-COVID-19 period covers a longer time span than the subsequent COVID-19 and post-COVID-19 intervals. Consequently, the observed rise in the relative proportion of voluntary cases may partly reflect a reduction in accidental drug poisonings rather than an actual increase in voluntary use. Nonetheless, the persistence of voluntary intoxications in adolescents remains a concern.

Based on our analysis and consistent with findings from other studies in the literature, there is an urgent and growing need for a coordinated, multidisciplinary endeavor among various healthcare professionals to address this healthcare issue. This collaborative initiative should launch a comprehensive awareness campaign to educate the public on the critical importance of “childproofing” common household items, including readily accessible medications, cleaning products, and cosmetics. Additionally, it is essential to monitor and address the risks associated with new and emerging substances, e.g., electronic cigarette liquids, which pose potential hazards in the home environment. Raising caregivers’ awareness about the safe storage and handling of these potentially dangerous substances is critical to prevent accidental poisonings and ensure the safety and well-being of children [35]. Such preventive measures require active engagement from pediatricians, pharmacists, toxicologists, and public health officials working together to promote effective strategies and policies.

It is also crucial to establish effective strategies for monitoring and supporting adolescents’ psychosocial well-being. These strategies should include training parents and teachers to recognize signs of emotional distress and enhancing psychological support services available to adolescents in schools, counseling centers, and online platforms [36]. Additionally, it is important to investigate the specific factors contributing to increased distress among adolescents, such as social isolation, academic difficulties, grief, and cyberbullying.

This work has some limitations that must be acknowledged. Firstly, the study’s retrospective design may limit accuracy. Retrospective studies are based on pre-existing medical records, which may introduce several potential biases. First, recall bias may affect the accuracy of the data because patients’ symptoms, treatments, and outcomes were recorded based on past events, and the level of detail may vary between cases. Additionally, some data may be incomplete or missing from medical records. The variable quality and completeness of data in medical records could introduce missing information and inaccuracies and limit the robustness of the analysis. Prospective evaluations could provide a more comprehensive overview of the general population.

Secondly, the absence of detailed data pertaining to socioeconomic status, family background, coexisting psychiatric conditions, and the implementation of preventive measures within the home environment significantly impedes a thorough and nuanced understanding of the various factors that may contribute to the occurrence of acute intoxication incidents. Moreover, this lack of comprehensive information limits the ability to accurately interpret and analyze the observed shifts in the patterns and trends of such events following the onset of the SARS-CoV-2 pandemic. Thirdly, our study was conducted in a single center, which does not enable a generalization of our findings to the entire pediatric population and limits the types of approaches and protocols analyzed to those used in our institution.

Finally, the lack of long-term follow-up data regarding late organic complications and psychological, domestic, and socio-familial implications limits a comprehensive assessment. This limitation precludes the ability to assess the long-term impact of acute events, as well as to identify predictive factors that may contribute to adverse outcomes over time. This underscores the paramount importance of undertaking future longitudinal studies, which are indispensable for monitoring patients over prolonged durations. Such investigations yield critical insights into the progression, potential complications, and risk factors associated with these acute events, thereby facilitating the development of more effective prevention, management, and intervention strategies.

## 5. Conclusions

Our analysis of pediatric intoxications during the Pre-COVID-19, COVID-19, and Post-COVID-19 periods revealed significant variations in incidence, modality, and substances involved. The home environment was confirmed as the primary location of exposure, with an increase in voluntary intoxications among adolescents, particularly in the post-pandemic period. This phenomenon may be related to the pandemic’s psychological impact, which was characterized by social isolation and emotional distress that encouraged alcohol and drug abuse.

The collected data highlight the need for targeted prevention strategies, such as raising awareness of home safety measures for younger children and improving access to psychological support services for adolescents. Further multicenter and longitudinal studies could provide a broader view of the long-term consequences of these phenomena, contributing to the development of more effective interventions for managing and preventing pediatric intoxication.

## Figures and Tables

**Table 1:** Characteristics of patients admitted to the PED for acute poisoning.

	N	%
**Age**		
0–5 years	512	64.48%
6–11 years	84	10.58%
12–18 years	198	24.94%
**Gender**		
Male	403	50.76%
Female	391	49.24%
**Environment**		
Home	502	63.22%
School	12	1.51%
Public place	67	8.44%
Juvenile detention centers	3	0.38%
Undefined	205	25.82%
**Time slot**		
0–6	146	18.39%
6–12	132	16.62%
12–18	218	27.46%
18–24	298	37.53%
**Intentionality**		
Accidental	607	76.45%
Voluntary	187	23.55%
of which		
* Anti-conservative*	*113*	*60.43%*
* Recreational*	*74*	*39.57%*
**Witnesses**		
Parents	562	70.78%
Siblings	10	1.26%
Other relatives	17	2.14%
Legal guardian	1	0.13%
Friends/peers	33	4.16%
None	27	3.40%
Other	144	18.14%
**Symptoms/Signs**		
Asymptomatic	300	37.78%
Nausea/vomit	185	23.30%
Pharyngeal/mucosal hyperemia	84	10.58%
Smell or traces of the substance	65	8.19%
Hypo-reactivity	46	5.79%
Numbness and sleepiness	42	5.29%
Abdominal pain	35	4.41%
Cough	24	3.02%
Oral cavity injuries	16	2.02%
Difficulty in breathing	16	2.02%
Burning mouth	13	1.64%
Diarrhea	13	1.64%
Conjunctival hyperemia	12	1.51%
Odynophagia/pharyngodynia	9	1.13%
Fever	8	1.01%
Sialorrhea	7	0.88%
Cutaneous hyperemia	4	0.50%
Tongue dysepithelialization	4	0.50%
Cold sweat	4	0.50%
Other	127	15.99%

**Table 2:** Contingency tables with analysis of acute intoxications from the Pre-COVID-19, COVID-19, and Post-COVID-19 periods in relation to age groups, gender of patients, intentionality of intake, and anti-conservative and recreational intent. The bold and underline are used to highlight statistically significant *p*-values.

	Pre-COVID-19		COVID-19		Post-COVID-19			
	N	%	N	%	N	%	*p*-Value (Chi-Square)	Cramér’s V
**Age**								
Pre-school age (0–5 years)	336	73.20%	76	58.91%	100	48.54%	**<0.00001 (61.4025)**	**0.17**
School age (6–11 years)	54	11.76%	11	8.53%	19	9.22%		
Adolescents (12–18 years)	69	15.03%	42	32.56%	87	42.23%		
**Gender**								
Male	234	50.98%	70	54.26%	99	48.06%	0.536854 (1.2441)	0.040
Female	225	49.02%	59	45.74%	107	51.94%		
**Intentionality**								
Accidental	402	87.58%	91	70.54%	120	58.25%	**<0.00001 (73.38)**	**0.30**
Voluntary	57	12.42%	38	29.46%	86	41.75%		
**Anti-conservative intent**								
Yes	47	10.24%	24	18.60%	42	20.39%	**0.000743 (14.4105)**	**0.13**
No	412	89.76%	105	81.40%	164	79.61%		
**Recreational intent**								
Yes	16	3.49%	14	10.85%	44	21.36%	**<0.00001 (54.1745)**	**0.26**
No	443	96.51%	115	89.15%	162	78.64%		

## Data Availability

The raw data supporting the conclusions of this article will be made available by the authors upon request.

## Supplementary Materials

The following supporting information can be downloaded at: https://www.mdpi.com/article/10.3390/medicina61091507/s1, Table S1: Types of substances responsible for acute intoxication in pediatric patients in terms of total sample incidence and incidence in the Pre-COVID-19, COVID-19, and Post-COVID-19 periods. Table S2: Substances used by the adolescent population for anti-conservative purposes. Table S3: Substances used by the adolescent population for recreational purposes. Table S4: Management of pediatric patients with acute intoxication.

## Abbreviations

The following abbreviation is used in this manuscript: PED—Pediatric Emergency Department.
