# Supplementary material for: Acute Poisoning in Children Presenting to the Pediatric Emergency Department: An Epidemiologic Study and the Impact of the SARS-CoV-2 Pandemic
Source: Medicina (Kaunas). 2025 Aug 22;61(9):1507. doi: 10.3390/medicina61091507 (PMC12471646; doi:10.3390/medicina61091507)
Supplement: Supplementary file 1 [file medicina-61-01507-s001.zip › medicina-3759164-supplementary.pdf]

Table S1. Types of substances responsible for acute intoxication in pediatric patients in terms of total sample incidence and incidence in the Pre-COVID, COVID, and Post-COVID periods.

	TOTAL		Pre-COVID		COVID		Post-COVID	
	N	%	N	%	N	%	N	%
**Drugs**	312	39.29%	201	43.79%	46	35.66%	66	32.04%
**Solvents**	141	17.76%	96	20.92%	21	16.28%	24	11.65%
**Alcohol**	62	7.81%	11	2.40%	11	8.53%	40	19.42%
**Detergents**	58	7.30%	42	9.15%	7	5.43%	9	4.37%
**Caustic acids**	35	4.41%	27	5.88%	5	3.88%	3	1.46%
**Narcotics**	26	3.27%	10	2.18%	6	4.65%	10	4.85%
**Natural products**	27	3.40%	7	1.53%	7	5.43%	13	6.31%
**Cosmetics and personal care**	25	3.15%	17	3.70%	4	3.10%	4	1.94%
**Toxic plants**	23	2.90%	16	3.49%	1	0.78%	6	2.91%
**Sanitizers**	21	2.64%	14	3.05%	1	0.78%	6	2.91%
**Toxic gases and vapors**	20	2.52%	8	1.74%	4	3.10%	8	3.88%
**Caustic alkalis**	19	2.39%	11	2.40%	3	2.33%	5	2.43%
**Cigarette parts, tobacco, and nicotine**	17	2.14%	13	2.83%	0	0.00%	4	1.94%
**Insecticides**	12	1.51%	9	1.96%	2	1.55%	1	0.49%
**Glues, paints, and inks**	11	1.39%	7	1.53%	3	2.33%	1	0.49%
**Metals**	8	1.01%	3	0.65%	2	1.55%	3	1.46%
**Fungi**	5	0.63%	2	0.44%	0	0.00%	3	1.46%
**Agriculture products**	2	0.25%	2	0.44%	0	0.00%	0	0.00%
**Other**	43	5.42%	23	5.01%	5	3.88%	15	7.28%

Table S2. Substances used by the adolescent population for anti-conservative purposes.

	TOTAL		Pre-COVID		COVID		Post-COVID	
	N	%	N	%	N	%	N	%
**Drugs**	78	69.03%	31	65.96%	18	75.00%	29	69.05%
**Solvents**	14	12.39%	7	14.89%	3	12.50%	4	9.52%
**Caustic acids**	7	6.19%	4	8.51%	2	8.33%	1	2.38%
**Detergents**	5	4.42%	5	10.64%	0	0.00%	0	0.00%
**Toxic gases and vapors**	4	3.54%	0	0.00%	1	4.17%	3	7.14%
**Caustic alkalis**	3	2.65%	2	4.26%	0	0.00%	1	2.38%
**Sanitizers**	2	1.77%	1	2.13%	0	0.00%	1	2.38%
**Natural products**	1	0.88%	0	0.00%	0	0.00%	1	2.38%
**Cosmetics and personal care**	1	0.88%	0	0.00%	0	0.00%	1	2.38%

Table S3. Substances used by the adolescent population for recreational purposes.

	TOTAL		Pre-COVID		COVID		Post-COVID	
	N	%	N	%	N	%	N	%
**Alcohol**	51	68.92%	8	50.00%	8	57.14%	35	79.55%
**Narcotics**	14	18.92%	5	31.25%	5	35.71%	4	9.09%
**Alcohol + narcotics**	9	12.16%	3	18.75%	1	7.14%	5	11.36%

Table S4. Management of pediatric patients with acute intoxication.

	N	%
**Transportation**		
Autonomous	133	16.75%
Ambulance	181	22.80%
Air ambulance	18	2.27%
Other	462	58.19%
**Triage urgency level**		
Low	167	21.03%
Medium	545	68.64%
High	82	10.33%
**Laboratory tests**		
No	378	47.61%
Yes	416	52.39%
**Radiology examinations**		
No	554	69.77%
Yes	240	30.23%
**Esophagogastroduodenoscopy**		
No	753	94.84%
Yes	41	5.16%
**Treatment**		
Antidote	12	1.51%
Gastric lavage	54	6.80%
Activated charcoal	121	15.24%
Gastric lavage + activated charcoal	38	4.79%
**Outcome**		
Discharge	654	82.37%
Admission	140	17.63%
Of which		
Intensive environment	*38*	*27.14%*
Non-intensive environment	*102*	*72.86%*
